# The Impacts of Programs and Policies to Address Food Insecurity: An Analysis of Change in Income

**DOI:** 10.3390/nu17010154

**Published:** 2024-12-31

**Authors:** Eva Nelson, Jacqueline Milton Hicks, Lok Hang Kristina Keung, Elizabeth Rhoads, Jemima Mascary, Jacey A. Greece

**Affiliations:** 1 Department of Community Health Sciences, Boston University School of Public Health, 801 Massachusetts Ave. 4th Floor, Boston, MA 02118, USA; ehnelson@bu.edu; 2 Department of Biostatistics, Boston University School of Public Health, 801 Massachusetts Ave. 3rd Floor, Boston, MA 02118, USA; jnmilton@bu.edu (J.M.H.); kkeung@bu.edu (L.H.K.K.); rhoadsel@bu.edu (E.R.); jcalixte@bu.edu (J.M.)

**Keywords:** food insecurity, income change, public health intervention, federal assistance programs, food banks, food pantries

## Abstract

Background/Objectives: This paper examines hunger over time to analyze how food insecurity is impacted by reduced income, including household funding from the government. Federal policies and community-based programs have the ability to prevent increases in food insecurity, particularly for populations that have risk factors, such as households with children; single-parent households; low-income households, especially those in rural areas; Black and Hispanic households; and, households experiencing economic hardships. Methods: This study is bas ed on a cross-sectional survey that was administered in 2018 and 2019 to food pantry clients, an already food insecure population accessing resources, in Eastern Massachusetts. Baseline surveys were matched with a 3-month follow-up survey (*n* = 308) and multinomial logistic mixed effect models were used to analyze the association between change in household hunger and change in household income. Results: This study found that participants who experienced decreased income compared to no income change from baseline to follow-up had 2.16 times the odds (95% CI: 1.05, 4.46) of experiencing increased household hunger compared to no change in hunger from baseline to follow-up, after adjusting for all other covariates. Conclusions: Food insecurity in the United States remained stable during the beginning of COVID-19, despite prevalence of reduced household income. The expanded government benefits that were implemented early in the pandemic contributed to total household income, which prevented increased food insecurity. Increased food insecurity after the removal of benefits starting in 2022 indicates the importance of continuing support established during times when consistent income is compromised to prevent a delayed rise in food insecurity.

## 1. Introduction

The COVID-19 pandemic’s onset caused many people to experience singular or cumulative economic hardships, which could have resulted in potential exacerbation of food insecurity [1]. However, during the height of the pandemic, food insecurity in the U.S. remained consistent at 10.2% between 2019 through 2021, partly due to federal government’s and community-based organizations’ (CBOs) responses to anticipated increases in food insecurity [2,3]. With the removal of these expanded benefits in 2022, which often dictate a portion of household income, 12.8% of households experienced food insecurity and it continues to rise with 13.5% households in 2023 [4].

The U.S. Department of Agriculture (USDA) defines food insecurity as the limited or uncertain ability to acquire nutritionally adequate and safe foods and hunger refers to an individual-level psychological condition that may result from food insecurity [4]. Certain demographics in the U.S. historically and during the pandemic have disproportionately higher rates of food insecurity compared to others [2,3] highlighting the need to address this health disparity [5]. Populations disproportionately impacted are households with children; single-parent households; low-income households, especially those in rural areas; Black and Hispanic households; and, households experiencing economic hardships [5,6,7,8] including unemployment, job loss, decreased income, family structure changes, lack of home ownership, eviction, or limited financial savings [5,9,10]. For example, non-Hispanic Black and Hispanic employees were least likely to have paid sick time, increasing economic hardships for a demographic already experiencing disproportionate food insecurity [10].

This paper examines the relationship between hunger and change in income over time to understand the impacts that financial hardships can have on food security. While this study specifically evaluates hunger, food insecurity is a key construct throughout this paper because it is a more widely used data point in research and is often used to indicate population-level determinants. The data presented is from a baseline and follow-up survey conducted in 2018 and 2019, respectively, among food pantry users. The findings of the survey are relevant to present day context and federal assistance supports. Additionally, this study population is already at risk for experiencing food insecurity, allowing intervention recommendations to be more targeted toward those who need resources the most. Covariates in this study included participant race, age, sex, education level, marital status, occupation status at baseline, and household size at baseline and presence of kids under 18 years of age in household, seniors 65 years or older in household, food assistance, and economic hardship.

Although this study was conducted in 2018 and 2019, the social determinants of food insecurity remained consistent and were exacerbated during the COVID-19 pandemic. The comprehensive approach of expanded government benefits with CBO mobilization likely prevented an increase in food insecurity during the height of the pandemic [2]. Food insecurity increased in 2022 and 2023, shortly after expanded government benefits were removed, which was particularly problematic for people experiencing financial hardships over time [4]. Findings from this study can inform future strategies for resource allocation and program design to proactively address food insecurity in populations most in need of additional supports.

## 2. Materials & Methods

### 2.1. Study Sample and Data Collection

This study, a collaboration between an academic research institution and a food bank servicing a large geographic area and a number of food pantries, assessed levels and determinants of hunger in Eastern Massachusetts communities through a cross-sectional survey administered to food pantry users who visited one of ten food bank pantries. A food bank stores and distributes food to food pantries, which then serves the distributed food to clients, in addition to any food donated directly to the food pantry through other mechanisms other than the food bank. Food banks typically have resources and mechanisms to store all food, including refrigerated and frozen food, and are open during normal business hours while food pantries often times are open select hours and days per week and for the purposes of immediate distribution and not food storage. Food pantry clients were considered eligible for the study if they (1) were at least 18 years or older; (2) were physically and mentally capable of completing the survey; (3) spoke English or Spanish; and, (4) were not planning on moving within the next three months.

The 15-min incentivized survey included questions on demographics, hunger, expenditures, resources, and economic hardships and was either self-administered on a tablet or interviewer-administered. Baseline surveys were conducted from June 2018 through August 2018 at high volume food pantries defined as those that served at least 1000 households per month in the prior year. From the 1444 pantry users approached for the study, 825 (57%) agreed to participate; met the eligibility criteria (were at least 18 years old, were physically and mentally capable of completing the survey, spoke either English or Spanish, were not planning to move within the next three months); and, provided verbal consent to participate in the study. Reasons for refusal to participate in the study included being in a rush and not having enough time, not speaking English or Spanish, and/or not understanding what constituted study participation. Follow-up surveys were conducted from September 2018 through February 2019—three to six months following the baseline survey. Approximately 74% (*n* = 442) of the 599 completed follow-up surveys were matched to baseline surveys. Participants with incomplete surveys were excluded from analyses resulting in 308 participants (51% of the follow-up sample). This study was reviewed and approved by the University’s Institutional Review Board (H-37567) and verbal informed consent was obtained and recorded.

### 2.2. Measures

The primary exposure of interest was self-reported household income after tax, calculated as change score from baseline to follow-up. Respondents were asked “What was your household’s total income last month after taxes?” with response categories ranging from “Less than $500” to “$3000 or more” increasing in $500 increments. Change in household income represented the difference from baseline to follow-up and categorized into (1) no change in income-level category, (2) increase in income-level category, and (3) decrease in income-level category from baseline to follow-up. The exposure variables were selected based on prior literature that shows associations between these factors and food insecurity [6,9,11].

The primary outcome of interest was change in household hunger from baseline to follow-up, assessed through a modified version of the validated Household Hunger Scale (HHS) [6]. The HHS requires participants to rate on a four-point scale how often in the past 30 days (1) there was no food to eat of any kind in their house because of lack of resources to get food; (2) the participant or any household member went to sleep hungry because there was not enough food; and, (3) the participant or any household member went a whole day and night without eating anything because there was not enough food. Response options included “never (0 times)”, “rarely (1–2 times)”, “sometimes (3–10 times)”, and “often (10+ times)”. A hunger indication score was calculated by summing the scores for each question. Change in household hunger represented the difference of the hunger indication scores from baseline to follow-up and categorized into (1) no change in hunger, (2) increase in hunger, and (3) decrease in hunger.

This analysis considered several covariates based on prior literature on the association between hunger and household income and were assessed for potential confounding. Covariates included participant race, age, sex, education level, marital status, occupation status at baseline, and household size at baseline and dichotomous yes/no presence of kids under 18 years of age in household, seniors 65 years or older in household, food assistance, and economic hardship.

### 2.3. Statistical Analyses

Demographic characteristics of the study cohort were compared across change in household income from baseline to follow-up categories. Means and standard deviations were computed for continuous demographics and frequencies and percentages were computed for non-continuous demographics. Standard errors were clustered by pantry site. Multinomial logistic mixed effect models were fitted to examine the association between change in household hunger (increased hunger vs. no change, and decreased hunger vs. no change) and change in household income to account food pantry site clusters. These models adjusted for pantry site as a random effect and all covariates were controlled for as fixed effects. *p*-values were determined to be statistically significant at an alpha of 0.05. Statistical analyses used SAS Studio.

## 3. Results

### 3.1. Participant Characteristics

Of the 308 matched respondents in the analytic cohort, 85 (27.6%) participants experienced a decrease and 82 (26.6%) experienced an increase in income from baseline to follow-up with the rest (141, 45.8%) reporting no change. Baseline characteristics between participants across the three categories were distributed similarly. The average age was 52.5 years (SD: 13.6) with the majority being female and having education of high school or some college (Table 1). Respondents who had no change in income from baseline to follow-up were more likely to be Non-Hispanic White (45.4%), more likely to be a one-member household (41.8%), more likely to have utilized SNAP assistance in the past thirty days, less likely to have kids aged 18 and under in the household (30.5%), and less likely to have utilized WIC assistance in the past thirty days (4.3%).

### 3.2. Adjusted Mixed Effect Models

This analysis used logistic mixed-effect models adjusting for covariates and clustering by pantry site. Participants who experienced decreased income, compared to no change in income, from baseline to follow-up had 2.2 times the odds (95% CI: 1.1, 4.5, *p* = 0.04) of experiencing increased more severe household hunger (“more hungry”) rather than no change in hunger, after adjusting for all other covariates. The same statistically significant relationship was not observed in those who experienced household hunger but to a lesser extent (“less hungry”) when income decreased from baseline to follow-up (OR: 0.7, 95% CI: 0.4, 1.2, *p* = 0.18) suggesting that those with a higher severity of hunger are more susceptible to being more hungry with decreases in income over time (Table 2). Other demographic characteristics demonstrated statistically significant associations in participants that were “less hungry” and “more hungry” compared to no change in hunger status from baseline to follow-up including having less than high school education compared to college graduate (*p* = 0.02 and *p* = 0.04, respectively) and not working compared to working full-time (*p* = 0.01 and *p* = 0.01, respectively).

## 4. Discussion

The food insecurity spike in 2022 to 2023 highlights the importance of initiating and continuing evidence-based programs and policies that reduce and prevent food insecurity [12]. To respond to expected food insecurity increase at the beginning of the COVID-19 pandemic, the U.S. federal government enacted the Coronavirus Aid, Relief, and Economic Security (CARES) Act to increase food-related and economic support for households experiencing hardships due to the COVID-19 pandemic [13]. The CARES Act was associated with reduction in food insecurity [13].

One component of the CARES Act was Emergency Allotments (EA), which expanded Supplemental Nutrition Assistance Program (SNAP) benefits for many households. EA was most impactful for non-Hispanic Black and Hispanic households, a demographic already disproportionately impacted by food insecurity [1,9,14]. Despite the efficacy of this government support, the federal government removed EA for all states in March 2023 and some states removed expanded benefits even sooner [1]. Consequently, 25% of households receiving SNAP benefits impacted by the end of EA reported “sometimes” or “often” not having enough to eat [15].

Under the CARES Act, the U.S. government also implemented financial support for households experiencing economic hardships during the COVID-19 pandemic. For example, Unemployment Insurance (UI) was associated with a 35% decrease in reporting food insecurity [13], illustrating the interconnected relationship between food insecurity and financial insecurity. However, UI was removed in September 2021.

Medicaid, which existed before the COVID-19 pandemic, is a joint federal and state program that helps cover the costs for some people with limited income and resources [16]. At the onset of the pandemic, healthcare spending plummeted, so Medicaid benefits were expanded in some states as another aspect of the CARES Act [16]. In states that did not expand Medicaid, the decline in spending on healthcare services declined at a greater rate compared to states that expanded Medicaid benefits, proving the efficacy of Medicaid expansion [16].

The CARES Act was successful in preventing an increase in food insecurity, supporting households that experienced job loss, and promoting continued healthcare spending. However, the removal of all of these programs coincided with an increase in food insecurity in the U.S. Removal of government financial support is particularly problematic because this study found that a reduction in income could cause an increase in hunger in a short time period. A pandemic should not be the catalyst to adequately support people experiencing food insecurity and a non-pandemic environment should not be the reason to remove resources that, absent a disaster state, are necessary to support people in need. Additionally, barriers for accessing food assistance programs that existed before the pandemic were exacerbated during it, including stigma, geographic inaccessibility, limited transportation, and lack of awareness of resources [11,17]. A comprehensive approach that involves government policies, such as food assistance programs, and CBO programs, such as food banks and food pantries, is needed to address food insecurity and its root causes. Sustainable changes to addressing food insecurity in a normal state will require a multi-sector approach aimed at all levels of influence for a household.

At the community level, food assistance programs mobilized during the pandemic to reach more clients resulting in 40% of food pantry clients in 2020 using those resources for the first time [18]; receipt of food from charitable assistance programs increasing by 50% among disadvantaged adults [19]; and, free or low-cost school breakfast and lunch programs being expanded [7,19]. Efforts to support households must come from all levels of environmental influence to be most effective.

This study found that decreased income had over two times the amount of household hunger over three months. Although this study was conducted before the pandemic, these results are timely due to sudden and prolonged household income decreases because of employment loss and reduced work hours prompted by the pandemic [2]. Unlike other studies, this study assessed change in income and hunger specifically among food pantry clients, a vulnerable population already accessing resources. By focusing research on this population, the results lead to more targeted interventions. Consistent with this study’s finding that decreased income causes an increase in hunger, recent data is showing that in less than six months after the removal of EA, there has been an increase in food insecurity among people who lost some of their expanded SNAP benefits, which contribute to total household income [15,17]. Future research should investigate the longer-term effects of determinants of hunger and the extent to which changes in those determinants affect hunger severity over time. Based on this study’s findings and longer-term outcomes of the pandemic, however, there are four recommendations for future interventions to comprehensively and proactively address food insecurity.

First is to address racial disparities in food security. Although food insecurity overall did not increase during the pandemic, food insecurity for Black and Hispanic people disproportionately increased [5,10]. Unemployment and lack of jobs, the root causes of low-income and therefore higher food insecurity rates, are more prevalent in Black and Hispanic communities [9]. Interventions to address these disparities include establishing opportunities for communities of color by advocating for more jobs, providing job training, investing in education, and improving transportation in communities of color [9,20].

Second is application for government benefits should be more accessible. Despite the stability of food insecurity during the COVID-19 pandemic, some research shows that uptake of federal assistance programs, such as SNAP and WIC, did not increase during that time [2]. The government can work with CBOs to make the SNAP enrollment process more straightforward and expand outreach to support application processes, thus targeting communities most impacted by food insecurity [21].

Third is to increase availability of and access to nutritious, diverse foods in a non-stigmatizing way. During the pandemic, food assistance programs responded to widespread decreased income by implementing curb-side or pick-up distributions and expanding mobile and home delivery services [11]. Improving access to nutritious foods for low-income households by bringing food directly to communities that would not otherwise have access is critical for addressing food insecurity [5,22].

Fourth is to involve multiple areas of public health in addressing food insecurity. The healthcare sector plays a critical role in screening for food insecurity and connecting individuals to existing resources [2]. Food pantries can support this by providing detailed nutritional information about the foods being served and train staff and volunteers to emphasize healthy, culturally appropriate diets [22].

Although a limitation of this study is that it was conducted in 2018 and 2019, the social determinants of food insecurity remained the same and were exacerbated during the COVID-19 pandemic, making the findings still relevant and useful for future planning. Additionally, recent food insecurity statistics from the USDA were used to contextualize the findings within the current landscape of food insecurity. This study was conducted in one state with a specific context, which was similar to factors and experiences across other states within the U.S., however some results may not be generalizable beyond the context of this study. As public health interventions should be tailored to the populations they support given the contextual factors influencing the problem and using evidence to inform solutions, the findings from this study serve to add to the research to support future practice and policy in this area to the extent they are applicable. A second limitation was the sample size and sociodemographic composition of food pantry users at each site varied and the level of food pantry services that pantries provided were not controlled for in analyses [23]. Third, recall bias and negative social perceptions of food insecurity may have impacted the quality of data collected from study participants [23].

## 5. Conclusions

Recent data supports the likely possibility that food insecurity in the U.S. did not increase during the height of the COVID-19 pandemic as a result of mobilization of CBOs and government response [15] though it still increased for certain demographics that are disproportionately affected [17]. Since the removal of EA in 2022, more households are experiencing food insecurity than during the beginning of the pandemic [17], a trend that is likely to continue. Designing and implementing comprehensive approaches that reach people with financial hardships will be most effective in equitably alleviating food insecurity.

## Figures and Tables

**Table 1:** Demographic characteristics by change in household income after tax from baseline to follow-up (Income increase vs. no change vs. income decrease) among matched survey participants, United States, 2018–2019.

	Change in Household Income from Baseline to Follow Up		
Characteristic	Income Decrease N = 85	No Change N = 141	Income Increase N = 82
**Age, mean (SD*)***	51.9 (13.2)	54.2 (13.6)	50.1 (13.9)
**Education Level at Baseline, *n (%)***			
Less than High School	21 (24.7)	30 (21.3)	16 (19.5)
High School or Some College	51 (60.0)	91 (64.5)	46 (56.1)
College Graduate (4 years) or More	13 (15.3)	20 (14.2)	20 (24.4)
**Race, *n (%)***			
Non-Hispanic White	26 (30.6)	64 (45.4)	27 (32.9)
Non-Hispanic Non-White	32 (37.7)	39 (27.7)	28 (34.2)
Hispanic	27 (31.8)	38 (27.0)	27 (32.9)
**Marital Status at Baseline, *n (%)***			
Single or Never Married	30 (35.3)	50 (35.5)	31 (37.8)
Separated/Divorced or Widowed	35 (41.2)	56 (39.7)	25 (30.5)
Married or Living with Partner	20 (23.5)	35 (24.8)	26 (31.7)
**Occupation Status at Baseline, *n (%)***			
Working full time (≥35 h/week)	14 (16.5)	10 (7.1)	21 (25.6)
Working part time (<35 h/week)	21 (24.7)	21 (14.9)	19 (23.2)
Not Working **	50 (58.8)	110 (78.0)	42 (51.2)
**Sex, *n (%)***			
Female	59 (69.4)	103 (73.1)	64 (78.1)
Male	26 (30.6)	38 (27.0)	18 (22.0)
**Household Size at Baseline, *n (%)***			
0 to 1 member	19 (22.4)	59 (41.8)	15 (18.3)
2 members	24 (28.2)	33 (23.4)	19 (23.2)
More than 2 members	42 (49.4)	49 (34.8)	48 (58.5)
**Kids (<18 years) in Household (Yes)**	40 (47.1)	43 (30.5)	46 (56.1)
**Seniors in Household (Yes)**	26 (30.6)	44 (31.2)	22 (26.8)
**SNAP Assistance Use in Past 30 Days**	46 (54.1)	87 (61.7)	45 (54.9)
**WIC Assistance Use in Past 30 Days**	8 (9.41)	6 (4.3)	8 (9.8)
**Food Pantry Use in Past 30 Days**	75 (88.2)	131 (92.9)	71 (86.6)
**Any Economic Hardship (Yes)**	48 (56.5)	84 (59.6)	54 (65.9)

** “Not Working” occupation status at baseline includes the following occupation categories: unemployed, disabled, retired, homemaker, and other.

**Table 2:** Association between change in household income after tax from baseline to follow-up and change in household hunger score from baseline to follow-up among matched survey participants, United States, 2018–2019.

	Less Hungry vs. No Change (*n* = 237)		More Hungry vs. No Change (*n* = 214)	
Variable	Adjusted Odds Ratio (95% CI)	*p*-Value	Adjusted Odds Ratio (95% CI)	*p*-Value
**Age (years)**	1.0 (0.9, 1.0)	<0.01	1.0 (0.9, 1.0)	0.15
**Monthly Baseline Household Income**				
*Income Decrease*	0.7 (0.4, 1.2)	0.18	2.2 (1.1, 4.5)	**0.04**
*Income Increase*	0.6 (0.3, 1.4)	0.26	1.3 (0.8, 2.0)	0.35
*No Change*	Ref		Ref	
**Education Level**				
*Less than High School*	1.7 (1.1, 2.7)	**0.02**	3.0 (1.1, 8.4)	**0.04**
*High School or Some College*	2.9 (1.2, 7.1)	**0.02**	1.6 (0.8, 3.1)	0.16
*College Graduate (4 Years)*	Ref		Ref	
**Occupation Status at Baseline**				
*Working Part Time (<35 h per week)*	0.8 (0.5, 1.2)	0.20	1.9 (0.7, 4.6)	0.20
Not Working **	0.4 (0.2, 0.8)	**0.01**	2.7 (1.3, 5.5)	**0.01**
*Working Full Time (>=35 h per week)*	Ref		Ref	
**Race**				
Non-Hispanic Non-White *	0.5 (0.2, 1.1)	0.10	0.7 (0.3, 2.1)	0.56
Hispanic *	1.2 (0.9, 1.7)	0.26	0.9 (0.4, 2.1)	0.75
Non-Hispanic White *	Ref		Ref	
**Marital Status**				
*Single/Never Married*	1.2 (0.8, 1.9)	0.35	0.5 (0.2, 1.2)	0.13
*Separated/Divorced/Widowed*	1.3 (0.67 2.5)	0.47	0.7 (0.30 1.6)	0.41
*Married/Living with Partner*	Ref		Ref	
**Sex**				
*Male*	1.1 (0.6, 2.1)	0.75	0.7 (0.4, 1.2)	0.16
*Female*	Ref		Ref	
**Household Size**				
*2 Members*	1.5 (0.5, 4.7)	0.51	0.6 (0.2, 1.5)	0.25
*3 or More Members*	1.8 (0.9, 3.5)	0.09	1.48 (0.8, 2.8)	0.22
*0 to 1 Member*	Ref		Ref	
**Household Composition**				
Kids (Under 18 years) in Household (Yes)	1.0 (0.4, 2.2)	0.97	1.3 (0.6, 2.9)	0.57
Seniors in Household (Yes)	0.9 (0.5, 1.6)	0.66	0.5 (0.4, 0.8)	**<0.01**
**Food Assistance Use in Past 30 Days**				
SNAP	1.0 (0.6, 1.6)	0.88	1.8 (1.1, 23.1)	**0.02**
WIC	0.8 (0.3, 2.7)	0.74	0.1 (0.00, 2.3)	0.13
Food Pantry Use	0.5(0.2, 1.3)	0.14	0.6 (0.4, 0.8)	**0.01**
**Any Economic Hardship (Yes)**	1.6 (1.1, 2.3)	**0.02**	1.2 (0.7, 2.1)	0.49

** “Not Working” occupation status at baseline includes the following occupation categories: unemployed, disabled, retired, homemaker, and other. * The U.S. Census’ use of the term race reflects the social definition of race that looks at peoples’ national origin or sociocultural groups. There are five racial categories: White, Black or African American, American Indian or Alaska Native, and Native Hawaiian or Other Pacific Islander. The term ethnicity refers to Hispanic or Latino origin and the two categories are: Hispanic or non-Hispanic. Some people may identify with multiple races or ethnicities.

## Data Availability

The data presented in this study are available on request from the corresponding author.
